# Effect of Indirect Pulp Capping Materials On Regional Dentin Seal

**DOI:** 10.3290/j.jad.c_2109

**Published:** 2025-06-19

**Authors:** Roland Frankenberger, Nora Michalowski, Stefanie Amend, Susanne Lücker, Norbert Krämer

**Affiliations:** a Roland Frankenberger Professor and Chair, Department of Operative Dentistry, Endodontics, and Pediatric Dentistry, University of Marburg, Medical Center for Dentistry, University Medical Center Giessen and Marburg, Campus Marburg, Georg-Voigt-Strasse 3, 35039 Marburg, Germany. Conceptualization, data curation, and manuscript writing.; b Nora Michalowski Dentist, Department of Operative Dentistry, Endodontics, and Pediatric Dentistry, University of Marburg, Medical Center for Dentistry, University Medical Center Giessen and Marburg, Campus Marburg, Georg-Voigt-Strasse 3, 35039 Marburg, Germany. Execution of experiments.; c Stefanie Amend Asisstant Professor, Department of Pediatric Dentistry, Medical Center for Dentistry, University Medical Center Giessen and Marburg, Campus Giessen, Schlangenzahl 14, 35392 Giessen, Germany. Data analysis.; d Susanne Lücker Research Fellow, Department of Pediatric Dentistry, Medical Center for Dentistry, University Medical Center Giessen and Marburg, Campus Giessen, Schlangenzahl 14, 35392 Giessen, Germany. Supervising experiments.; e Norbert Krämer Professor and Chair, Department of Pediatric Dentistry, Medical Center for Dentistry, University Medical Center Giessen and Marburg, Campus Giessen, Schlangenzahl 14, 35392 Giessen, Germany. Idea, proofreading.

**Keywords:** adhesive bond strength, dentin adhesives, lining, microtensile bond strength, surface characteristic

## Abstract

**Purpose**: The aim of this *in-vitro* study was to evaluate the effect of different indirect pulp capping (IPC) materials on bond strength to surrounding dentin.

**Materials and Methods:**

Fifty-six human third molars were used in this study. Occlusal dentin of 42 teeth was exposed. Dentin surfaces (n = 6) were left uncovered (control) or received a 1 × 1 mm central IPC (KL: Kerr life, DY: Dycal, TC: Theracal LC, CL: Calcimol LC, BD: Biodentine, and PR: ProRoot MTA) and were then bonded with Scotchbond Universal adhesive and restored with a composite resin build-up (Filtek™ Z250). After 24 h of water storage, the specimens were cut into sticks, which were marked red (1 mm distance from IPC spot), green (2 mm distance), and blue (3 mm distance). Consequently, µ-TBS tests were performed and analyzed using one-way ANOVA (P < 0.05) for normal distributions and Mann–Whitney U-test (P < 0.05) for non-normal distributions. Pretesting failures were recorded as 0 MPa. Fracture modes were analyzed under a fluorescence microscope, and interfaces and surfaces of 14 additional specimens were visualized under a scanning electron microscope (SEM).

**Results:**

A significant reduction in peripheral seal was only observed for KL (Mann–Whitney U-test, P < 0.05). All groups showed increasing bond strengths from the IPC area to the periphery, indicating a certain contamination potential of IPC materials.

**Conclusion:**

IPC materials being applied in very deep cavity areas except Kerr Life do not harm peripheral seal to dentin. Especially, hydraulic cements can be used without a negative effect on the peripheral dentin seal.

Direct and indirect tooth-colored restorations are routinely bonded to both enamel and dentin today.^27^ Successfully conditioned enamel is still the primary source of micro-retention, and additional dentin bonding helps to stabilize the remaining tooth and seals the dentin in order to protect pulp vitality and to avoid postoperative hypersensitivities.^17,27^ Conventional cement liners beneath composite resins are actually not routinely applied because they considerably reduce the bonding area, and moreover, they are inconvenient and time-consuming without providing additional value for the patient.^11,26^ However, these liners still play a significant role when cavities are estimated to be very deep and measures are taken to protect tooth vitality.^1,5,24^ In general, an effective dentin seal still represents the best way to protect vital pulp tissue,^7,20^ but with a remaining dentin thickness of < 300 µm it was reported that dentin permeability dramatically increases^19^ and odontoblasts may suffer reduced biomineralization performance under the direct influence of monomers such as TEGDMA9. So, for these extreme cases, deep cavity liners or conventional cement liners are still making sense.^2,19,21^ In most relevant papers, this phenomenon is referred to as indirect pulp capping (IPC).

The aim of the present *in-vitro* study was therefore to evaluate the influence of different regionally applied IPC materials and their contamination potential on dentin bond strength to surrounding dentin areas. This has already been previously shown for primary teeth6. The null hypothesis of the present investigation was that cavity liners do not harm dentin bonding to remaining dentin.

## MATERIALS AND METHODS

After obtaining ethical approval from the University of Giessen Ethics Committee (Germany, Code 143/09), 56 freshly extracted, caries-free third molars were stored in 0.5% chloramine T for less than 4 weeks. Teeth were ground flat to expose caries-free dentin, simulating caries excavation with a smear layer (Grinder-Polisher Beta, Buehler, USA) having been produced with abrasive paper under continuous water cooling (Met II (Grit 360 (P600) and Grit 600 (P1200), Buehler). Control specimens were bonded with Scotchbond Universal adhesive (Table 1). Test specimens received a central 1 mm × 1 mm IPC (Table 2), which was set and then bonded with the same adhesive. A composite resin build-up (Filtek™ Z250/3M, Table 3) was applied in 1 mm layers (Fig 1), each one light-cured for 40 s (bluephase® G2, Ivoclar Vivadent, Schaan, Principality of Liechtenstein). After 24 h storage in distilled water (37°C, Unity^TM^ Lab Services, Thermo Fisher Scientific, Waltham, USA), the samples were cut in sticks with a surface area of 0.5 mm^2^ (Fig 1, Isomet 5000 Linear Precision Saw, IsoMet^TM^ Diamond Wafering Blades (0.4 mm), Buehler; 3450 rpm, 2.5 mm/min, 75 g). After cutting, a hedgehog of still wax-fixed sticks with one central and three peripheral rows resulted. The sticks 1 mm away from IPC received red-color paintings, 2 mm were marked green, and 3 mm were painted blue. So each color represented a different distance from the IPC spot. Finally, 1170 sticks with no significant difference in sample size of different colors were subjected to µ-TBS tests (TC-550, Version 3.1.0.127, Syndicad, München).

**Table 1:** Adhesive systems used

Phosphoric acid	Composition	pH	LOT
Adhesive	Composition	pH	LOT
**DeTrey Conditioner 36** Dentsply Sirona, Konstanz, Germany	36% orthophosphoric acid, siliciumoxide, pigments, water	<2	2010000659, 2101000820
**3M** ^TM^ **Scotchbond** ^TM^ **Universal** Solventum, Seefeld, Germany	Bisphenol A-glycidyl methacrylate (Bis-GMA), hydroxy ethyl methacrylate (HEMA), phosphoryl methacrylate, water, ethanol, silica gel, polymeric acid, campherquinone, aromatic amine, methacrylic amine, butyl hydroxy toluol (BHT), 10-methacryloyloxidecyl dihydrogen phosphate monomer (10-MDP)	2.7	6756063, 6752814, 8113907, 7979849, 8540777, 8830691


**Table 2:** IPC materials used in the study

IPC material	Composition	Application	LOT
**Kerr Life Fast Set** Kerr, Herzogenrath, Germany	**Base:** N-Ethyl-o/p-toluolsulfonamide, calcium hydroxide, Zinc oxide **Catalyst:** Methyl salicylate, barium sulfate, titan dioxide, 2,2-Dimethyl propan-1,3-diol	Mix base and catalyst 1:1, apply with probe, wait 2:30 min	**Catalyst:** 8358510 **Base:** 8365153
**Dycal Dentin** Dentsply Sirona, Konstanz, Germany	**Base:** Disalicylate ester 1,3-butylen glycol, Zinc oxide, ferric oxide, calcium phosphate, calicium tungstenate **Catalyst:** Calcium hydroxide, ethyl toluol sulfon amid, zinc sterate, titanium dioxide, Zinc oxide	Mix base and catalyst for 10 s, apply with probe, wait 2:30 min	**Catalyst:** 80337 **Base:** 80338
**TheraCal LC®** BISCO, Schaumburg, IL, USA	Portland cement, Bisphenol A-glycidyl methacrylate (Bis-GMA), barium zirconate	Apply Calcimol LC with probe, light-cure for 20 s	2100006597
**Calcimol LC** VOCO, Cuxhaven, Germany	Urethandimethacrylate (UDMA), Triethylendimethacrylate (TEGDMA), calcium hydroxide, 2–dimethyl amino ethylmethacrylate, BHT	Apply Calcimol LC with probe, light-cure for 20 s	2148135
**Biodentine** ^TM^ Septodont, Niederkassel, Germany	**Powder:** Tricalcium silicate, Zirconia oxide, calcium oxide, calcium carbonate, ferric oxide pigments **Liquid:** Calcium chloride, polycarboxylate	1. Open Capsula 2. Apply 5 drops of liquid 3. Close capsula 4. Mix 30 s 5. Apply mixture on dentin 6. Wait 12 min	**Powder:** B28323 **Liquid:** B28365
ProRoot® MTA, Dentsply Sirona, Konstanz	Portland cement Calcium oxide, aluminum oxide, ferric oxide, calcium sulfate	Mix powder and liquid 1:1 for 1 min, apply with probe, wait 5 min	**Powder:** 289423 **Liquid:** 210218


**Table 3:** Buildup composite resin

Composite resin	Composition	LOT
**Filtek™ Z250 A3** Solventum, Seefeld, Germany	Ceramic, 3-(trimethoxysilyl)propyl methacrylate, bisphenol-A-polyethylenglycol-diether-dimethacrylate, dimethylacrylate, bisphenol-A-diglycidylmethacrylate, 2,2’-ethylen dioxy diethyl dimethacrylate, aluminumoxide	NA35856, NA87531, NE20203, NC94317, NE31043, NE67430, NE50366, NF31243, NE50366, NE20104, NF30469


**Fig 2:**
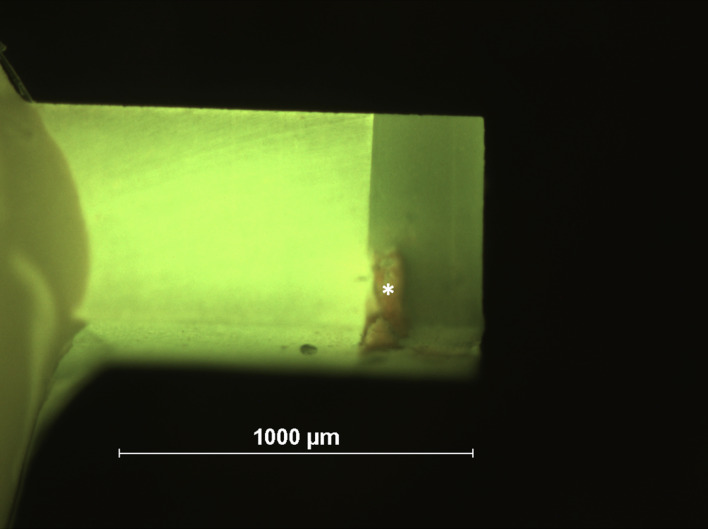
Fractography and filtering of IPC-contaminated specimens (IPC: asterisk) was performed using fluorescence microscopy.

After debonding, fractures were analyzed under a fluorescence microscope at 40× magnification (Nikon AZ100, Tokyo, Japan; Fig 2) for fracture mode (pre-test failure, cohesive in composite, adhesive, cohesive in dentin, mixed adhesive and dentin, mixed adhesive and composite, mixed composite and dentin) and remaining IPC materials which lead to exclusion of these specimens (Fig 2). Additional specimens were made to view the IPC effect under a scanning electron microscope (SEM) (Figs 3 and 4). Additionally, selected specimens were immersed in 4% sodium hypochlorite (diluted from 12% NaOCl, Carl Roth) for 20 min and demineralized in 20 % hydrochloric acid (diluted from 37% HCl, Sigma-Aldrich) for 6 h, each step followed by rinsing with distilled water. Afterwards, specimens were dehydrated in ascending concentration of ethanol (70% – 80% – 90% for 20 min, 100% for 1 h) and 1,1,1,3,3,3-hexamethyldisilazane for 10 min followed by drying overnight. The specimens were sputter-coated with gold (Sputter Coater, Polaron, SC502, Fisons Instruments, Ipswich, UK) and consequently examined under an SEM (SEM Amray Model 1610 Turbo, Amray, Bedford, MA, USA; Figs 3 and 4).

Statistical analysis was performed by SPSS® 26 (IBM®). Normal distribution was determined with the Kolmogorov–Smirnov test. One-way ANOVA test (P < 0.05) for normal distributions and Mann–Whitney U-test (P < 0.05) for non-normal distribution was used. The fracture analysis was performed descriptively. Therefore, the different fracture modifications were divided into three groups: pretesting failures (0 MPa), adhesive fractures, and cohesive fractures, which contain fractures within the composite resin, dentin, or mixed fractures.

## RESULTS

**Fig 5:**
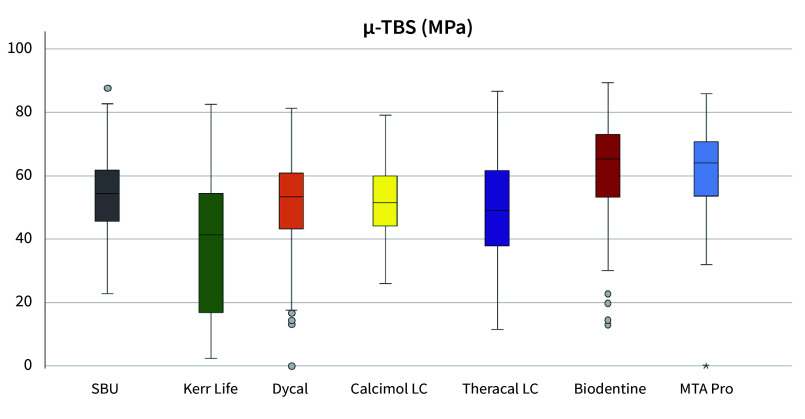
Boxplots of pooled microtensile bond strengths. Kerr Life caused significantly lower bond strength to the surrounding dentin.

**Fig 6:**
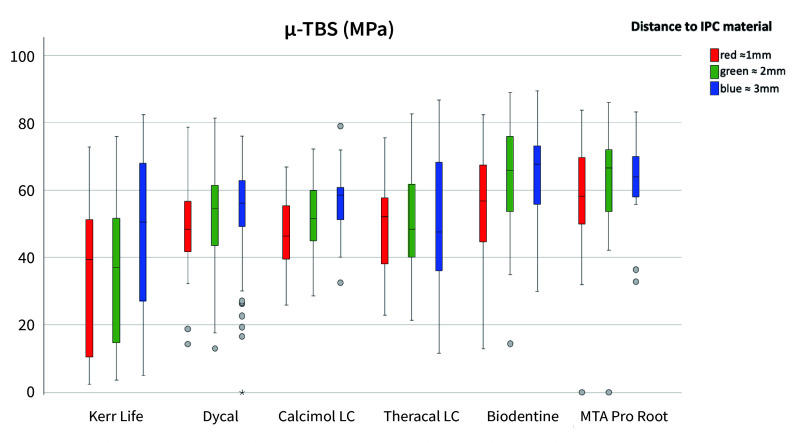
Boxplots of microtensile bond strengths relating to distance to IPC materials: Red: 1 mm, green: 2 mm, blue: 3 mm distance.

The results of the synoptic microtensile results are given in Figure 5. Regional bond strengths revealed that in red areas, ie, directly adjacent to the IPC area, Kerr Life showed a significantly negative influence on dentin bond strengths (post hoc, Bonferroni, P = 0.00, Fig 6). In green areas around IPC materials, adhesion values were again significantly lower in the Kerr Life group compared to all other CP materials (post hoc, Bonferroni, P = 0.00, Fig 6). Here, significantly higher adhesion values were found in Biodentine and MTA Pro Root groups compared with the other CP materials (post hoc, Bonferroni, P < 0.05, Fig 6). MTA Pro Root and Biodentine did not significantly differ in terms of adhesion (post hoc, Bonferroni, P = 1.000, Fig 6). When comparing all blue groups, adhesion values were significantly lower in Kerr Life compared to Biodentine (post hoc, Bonferroni, P = 0.000, Fig 6) and MTA Pro Root (post hoc, Bonferroni, P = 0.000, Fig 6). The Biodentine group exhibited significantly higher adhesion values than Kerr Life, Dycal, and Theracal LC groups. MTA Pro Root group showed significantly higher microtensile bond strength compared to Kerr Life and Theracal LC (post hoc, Bonferroni, P < 0.05, Fig 6).

When comparing pooled adhesion values between groups without distinct consideration of IPC distance, significantly lower microtensile bond strengths were found for Kerr Life compared to all other IPC materials and the control group (post hoc, Bonferroni, P = 0.000). Dentin bonding around Dycal was significantly lower than SCU (P = 0.009) and MTA Pro Root (P = 0.018). Peripheral adhesion around MTA Pro Root was higher than around Theracal LC (P = 0.018).

**Fig 7:**
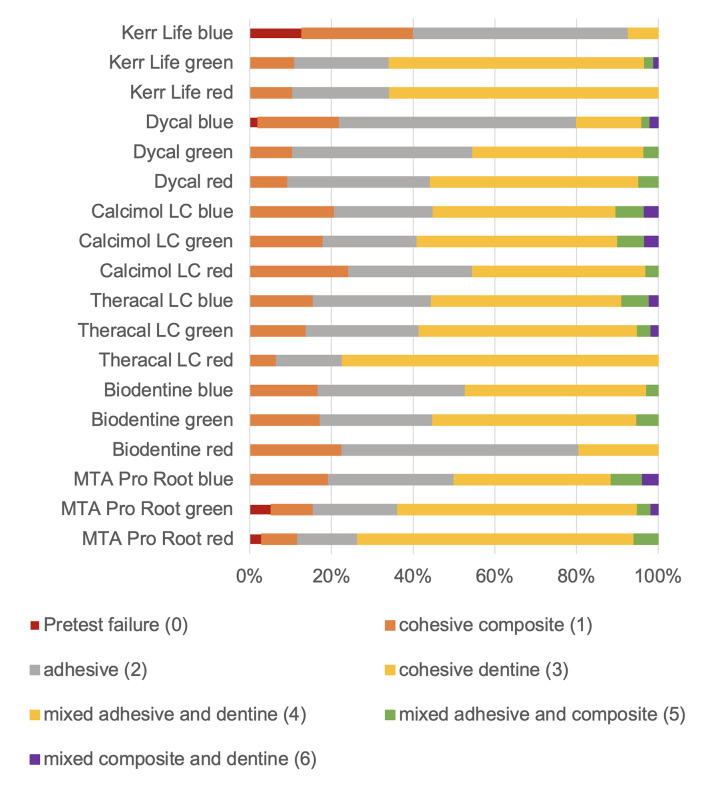
Results of fractographic investigation under fluorescence microscopy.

Fractographic results are displayed in Figure 7. Here, significantly more adhesive microtensile test failures were found around the IPC materials Kerr Life (blue), Dycal (blue), and Biodentine (red). The SEM analysis on representative samples could illustrate potential contaminations (Fig 4), but the detected traces under the SEM were not significant or linked to specific quantitative data. The interfacial analysis, including the measurement of hybrid layer thickness, was limited due to the cutting plane and direction, and due to the fact that only small amounts of interfaces were measured; also, no significant finding was recorded (Fig 3).

## DISCUSSION

IPC materials can be helpful for protecting pulp vitality in very deep cavities.^19,21^ The first and maybe most important question in this context is: Why should we still remove caries in the old-fashioned, aggressive way, which is today regarded as obsolete?^15,22^ When innovative caries excavation is carried out, characterized by the selective removal of carious tissue, we normally do not encounter ultra-thin remaining dentin thicknesses at the floor of the cavity without any necessity for IPC. So, one important imperative in the course of the present *in-vitro* study may be that these situations are primarily avoided by adopting a more conservative caries excavation protocol, which does not lead to remaining dentin thicknesses of > 30.^8^

Independent of the individual way of caries excavation, it would not be desirable when IPC materials would be on one hand effective vital pulp treatment *per se*, but on the other hand, would simultaneously corroborate an effective seal of surrounding dentin,^6^ which in turn is an important factor for the protection of tooth vitality.^3^ A previous investigation of primary tooth dentin revealed that exactly this may be the case.^6^ Astonishingly, the observed reduction of bond strength was not regionally different; it was found in all areas of the cavities up to the very periphery.^6^ So, at least for deciduous teeth, it could be clearly shown that these IPC liners represent a massive contamination of the whole bonding process.^6^

In general, it seems feasible that some of these rather soft IPC materials may not only release ions^14,24^ but also get superficially dissolved^18^ and therefore mix with the applied adhesive, which in turn may lose chemical effectiveness.^25^ Due to potential contamination reasons, the use of soft subbases is regarded as obsolete today; however, market data clearly show that they are still widely used – therefore, we included them in our study setup. Due to the fact that the chosen lining materials are of different chemical nature, it seems to be probable that they also differently affect dentin bonding in the surrounding dentin areas.^6,25^ Up to now, this phenomenon has not been found at all in the literature in the field, besides the previously mentioned study on deciduous teeth, where all materials under investigation affected dentin bond strength to remaining dentin.^6^

It is important to emphasize that in this study, the bond strengths of IPC materials have not been evaluated like, eg, in the similar-sounding study of Akin et al,^1^ because the bond strength of IPC materials is normally not measurable in a microtensile setup. We solely investigated dentin bond strength in the IPC periphery in order to detect any detrimental effect of these liners on dentin sealing quality around them. Therefore, we chose a setup with a stick diameter of < 1 mm in order to guarantee that IPC areas were really cut accordingly. Additionally, any resin-dentin stick exhibiting remnants of IPC was excluded from the measurements (Fig 2). In most of the cases, a gradient of bond strength values from the lining to the periphery was measured, indicating that IPC reveals a significant contamination effect. On the other hand, there have been materials that apparently did not affect peripheral dentin sealing and should therefore be preferred over the others. Finally, when indirect pulp capping materials do not provide any dentin bond strength at all, they may get loose when the adhesive is air-thinned in the course of adhesive cavity pretreatment, so a minimum dentin bond strength of these materials is also important from the clinical perspective. When investigating regional differences in dentin seal, the microtensile setup is the only one that really allows for evaluating the influence of different IPC materials, as proven in a previous publication.^6^ Nevertheless, the data scatter also shows that the focus on regional differences particularly shows some variations; however, the final results and conclusions are quite clear.

Also, the application mode of these lining materials may be of interest because there is the possibility of an immediate vs delayed bonding, which was already the subject of an *in-vitro* study.^4^ During the present investigation, we strictly stuck to the manufacturers’ recommendations regarding setting times; however, modified protocols may also be worthy of investigation in the future.

Another crucial aspect in this discussion is whether any of the investigated IPC materials really help to protect tooth vitality. A recent paper investigating clinical outcome challenges the claim that these very deep areas have to be covered with liners because a group without lining performed equally compared to the groups with different liners. Moreover, the European Society of Endodontics also recommends covering very deep areas, but it also lists glass-ionomer cement as a clinically recommended material for deep areas. So finally, the question remains whether there just has to be a blocker in order to prevent resin tags as mechanical disruption of vital pulp cells, or do we really need bioactive, ion-releasing biomaterials for pulp survival after deep carious decay?^16,23^ Finally, light-curing materials such as Calcimol LC and Theracal LC may, on one hand, not lower the bond strength of afterwards bonded composite resins; however, they are controversially discussed due to their monomer content.^10,13^ These materials have been included due to their market availability, rather than because the authors are convinced that they perform best for IPC.

Therefore, within the limits of this *in-vitro* study, it can be concluded that: (a) A more defensive, conservative caries excavation approach should be adopted in order not to face the investigated problem at all; (B) When doing so, a strict indication should be taken into consideration; and (c) Cavity lining materials such as Biodentine or ProRoot MTA should be preferred over calcium-salicylate containing, rather soft liners, which apparently are more prone to dissolve and cause contamination of remaining dentin areas. The null hypothesis was partially rejected.

### Clinical Relevance

In order to achieve the best possible peripheral dentin seal, IPC should be preferably carried out using hydraulic calcium silicate cements such as Biodentine or ProRoot. Soft subbases such as Kerr Life were shown to reduce dentin seal.

**Fig 1a to m:** Methodological setup.

**Fig 4a to e:** SEM surfaces of Calcimol LC (unbonded, a) vs bonded surfaces after bonding over Calcimol LC (b), Theracal LC (c), Dycal (d), Kerr Life (e), and Biodentine.

**Fig 3a to g:** SEM interfaces of control (a) vs dentin around Kerr Life (b), Dycal (c), Theracal LC (d), Calcimol (e), Biodentine (f), and ProRoot (g).






